# Behavioral and Neurochemical Consequences of Pentylenetetrazol-Induced Kindling in Young and Middle-Aged Rats

**DOI:** 10.3390/ph10030075

**Published:** 2017-09-13

**Authors:** Alexandre Ademar Hoeller, Cristiane Ribeiro de Carvalho, Pedro Leite Costa Franco, Douglas Affonso Formolo, Alexandre Kracker Imthon, Henrique Rodighero dos Santos, Ingrid Eidt, Gabriel Roman Souza, Leandra Celso Constantino, Camila Leite Ferreira, Rui Daniel Prediger, Rodrigo Bainy Leal, Roger Walz

**Affiliations:** 1 Graduate Program in Medical Science, Center of Health Sciences, University Hospital, Federal University of Santa Catarina, Florianópolis 88040-970, SC, Brazil; constantinolc@gmail.com (L.C.C.); rogerwalz@hotmail.com (R.W.); 2 Center of Applied Neuroscience (CeNAp), University Hospital, Federal University of Santa Catarina, Florianópolis 88040-970, SC, Brazil; decarvalhocr@gmail.com (C.R.d.C.); pedro.lcf@gmail.com (P.L.C.F.); douglasformolo@gmail.com (D.A.F.); alexandrekracker@gmail.com (A.K.I.); henrodsantos@gmail.com (H.R.d.S.); ingrideidt@hotmail.com (I.E.); gabrielromansouza@gmail.com (G.R.S.); rbleal@gmail.com (R.B.L.); 3 Department of Clinical Medicine, Center of Health Sciences, University Hospital, Federal University of Santa Catarina, Florianópolis 88040-970, SC, Brazil; 4 Graduate Program in Neuroscience, Center of Biological Sciences, Federal University of Santa Catarina, Florianópolis 88040-970, SC, Brazil; 5 Graduate Program in Health Sciences, Health Sciences Unit, University of Southern Santa Catarina, Criciúma, SC, 88806-000, Brazil; cami-leitte@unesc.net; 6 Department of Pharmacology, Center of Biological Sciences, Federal University of Santa Catarina, Florianópolis 88049-900, SC, Brazil; 7 Department of Biochemistry, Center of Biological Sciences, Federal University of Santa Catarina, Florianópolis 88049-900, SC, Brazil

**Keywords:** epilepsy, aging, anxiety, memory, pentylenetetrazol

## Abstract

(1) Objectives: Epilepsy disorder is likely to increase with aging, leading to an increased incidence of comorbidities and mortality. In spite of that, there is a lack of information regarding this issue and little knowledge of cognitive and emotional responses in aging subjects following epileptogenesis. We investigated whether and how aging distress epilepsy-related behavioral and biochemical outcomes are associated with cognition and emotion. (2) Methods: Young and middle-aged Wistar rats (3 or 12 months old) were treated with pentylenetetrazol (PTZ, 35 mg/kg) and injected on alternated days for 20 (young rats) and 32 days (middle-aged rats). Kindling was reached after two consecutive stages 4 plus one stage 5 or 6 in Racine scale. Control and kindled rats were evaluated in the elevated plus-maze (EPM) and object-recognition tests and their hippocampus was collected 24 h later for mitogen-activated protein kinases (MAPK) dosage. (3) Results: Middle-aged rats presented a higher resistance to develop kindling, with a decrease in the seizure severity index observed following the 4th and 9th PTZ injections. Middle-aged rats displayed an increased duration of the first myoclonic seizure and an increased latency to the first generalized seizure when compared to younger rats. The induction of kindling did not impair the animals’ performance (regardless of age) in the object-recognition task and the EPM test as well as it did not alter the hippocampal levels of MAPKs. (4) Significance: Our findings reveal that, despite age-related differences during epileptogenesis, middle-aged rats evaluated after kindling performed similarly during discriminative learning and emotional tasks in comparison to young animals, with no alteration of hippocampal MAPKs. Additional investigation must be carried out to explore the electrophysiological mechanisms underlying these responses, as well as the long-term effects displayed after kindling.

## 1. Introduction

Epilepsy is characterized by a persistent brain susceptibility to unprovoked seizures, occurring after at least one epileptic seizure [1]. This chronic disorder affects approximately 50 million people worldwide, deeply disturbing their quality of life, with an occurrence of about 48 per 100,000 cases per year in the United States [2]. Interestingly, the incidence of epilepsy is age-related and varies throughout lifespan, mainly affecting infants and middle-aged people (>50 years old). Hence, age- and epilepsy-related comorbidities (i.e., neurobiological, cognitive, psychological, and social impairment) are highly prevalent and also represent a clinical challenge [1].

Despite its high incidence, little is known regarding the neurobiological and clinical aspects, particularly the cognitive and emotional responses, displayed by people with late-onset epilepsy [3]. Such unpredictable scenarios impact preventive and therapeutic strategies that could significantly improve the prognosis in these unwell investigated patients [3]. Alternatively, specific features between young and aged brain function facing epileptogenesis in animal models may shed light into the mechanisms underlying this uncharted phenomenon and potentially translate to preventive and substitute therapeutic approaches [4].

The chemical kindling induced by pentylenetetrazole (PTZ), a GABA-A receptor antagonist, is a model of limbic epilepsy and is characterized by a persistent increase in seizure liability, with minimal neuronal injury and an absence of spontaneous recurrent seizures [5]. Interestingly, the behavioral impairments observed in rats following kindling is age-dependent, although further investigation is necessary to elucidate the ontogenetic aspects of epilepsy and its comorbidities [6]. Aged rats (26 months old) present an impaired spatial memory performance and a higher resilience following electrical hippocampal kindling, which may be reflected by aging effects on synaptic transmission efficacy [7]. Further, similar to young (3 months old) and different from aged-rats (28 months old), middle-aged rats (18 months old) display a sustained response to seizure activity with mild regenerative capacity when the hippocampal expression of GAP-43, a neuronal plasticity-related protein, is measured, whereas aged rats totally lost this competency [8].

Moreover, the synaptic plasticity, epileptogenesis, and seizure resilience are deeply influenced by intracellular enzymatic signaling [9]. The involvement of mitogen-activated protein kinases (MAPKs), a family of serine-threonine kinases that amplify and integrate signals triggered by a wide range of extracellular stimuli and regulate cell differentiation, survival and death [10], was only recently associated with complex and nonlinear modifications of the epileptogenic synaptic network in different animal models of epilepsy [11,12]. In spite of that, the putative aging effects in association with MAPKs activity, resilience to seizures and behavioral consequences remain to be explored.

In this sense, we investigated whether and how aging may alter epilepsy-related behavior and hippocampal biochemical (total and phosphorylated ERK1/2, JNK p54/p46 and p38) outcomes associated with learning, memory, and emotionality in middle-aged rats following a kindling protocol induced by the systemic injection of PTZ.

## 2. Results

### 2.1. Behavioral Seizure Threshold during Kindling Induction

**Figure 1:**
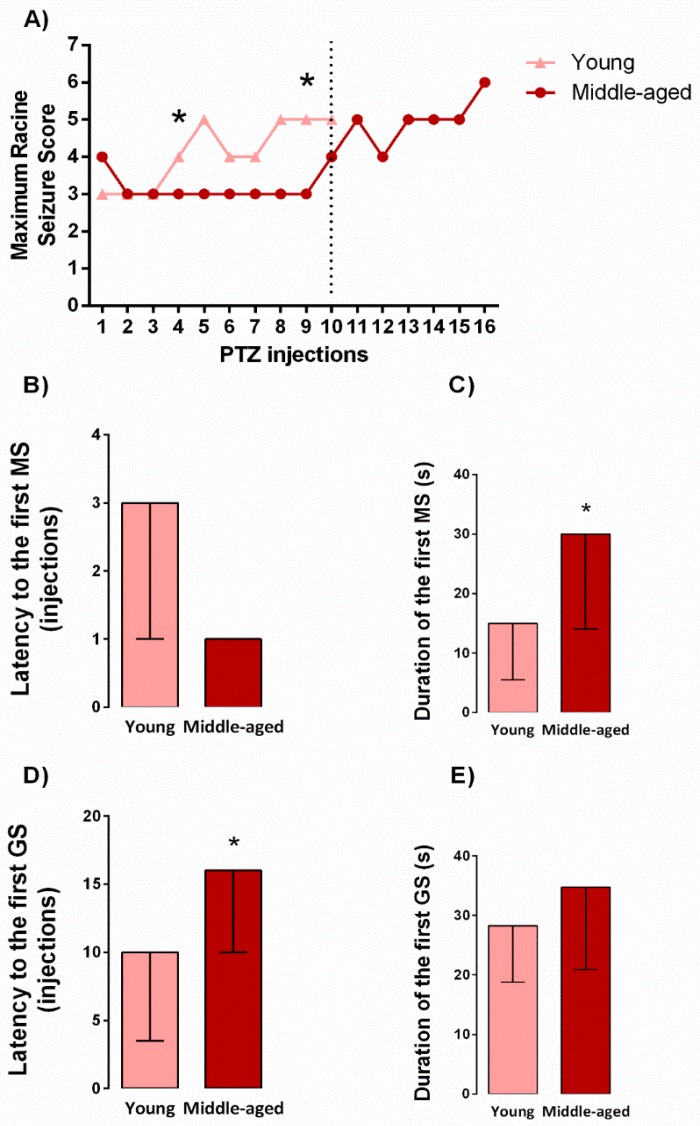
Effects of aging in the maximum racine seizure score during kindling progression. Dashed line delimits the number of injections applied to young rats (**A**). Latency and duration to the first myoclonic (MS) and generalized (GS) seizure (**B**–**E**). Values are represented by the median followed by Kruskal-Wallis ANOVA (**A**) or median ± interquartile range followed by Mann-Whitney U test (B–E). * *p* < 0.05 in the comparison between kindled groups (*n* = 11–17/group).

To better investigate the effects of aging on epileptogenesis induced by kindling, young and middle-aged rats were injected with PTZ, as previously described (see protocol design in Figure 5). As shown in Figure 1A, the middle-aged rats presented a higher resistance along kindling protocol when compared with younger following the 4th (H_1,28_ = 4,69, *p* = 0.03) and 9th (H_1,28_ = 4,50, *p* = 0.03) PTZ injections. Further, as revealed by the Mann-Whitney test, middle-aged rats displayed an increased duration of the first myoclonic seizure (U = 46, *p* = 0.03, Figure 1C) and an increased latency to the first generalized seizure (U = 32, *p* = 0.004, Figure 1D) during kindling in comparison to young rats.

### 2.2. Anxiety-Like Behavior Following Kindling Induction in Middle-Aged Rats

**Figure 2:**
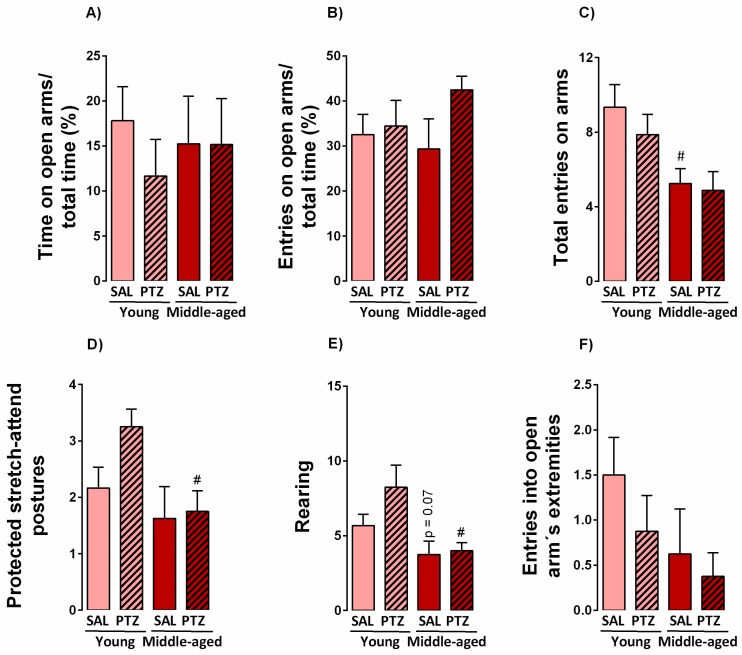
Effects of aging on anxiety-like behaviors of aged kindled rats evaluated in the elevated plus-maze test. Values are represented by the mean + S.E.M. followed by two-way ANOVA (A) and Newman-Keuls post-hoc test when required. ^#^ *p* < 0.05 in the comparison between kindled groups (*n* = 8–12/group).

To determine the effects of kindling on anxiety-like responses of middle-aged rats, we examined the behavioral profile of kindled rats in the elevated plus-maze test. Two-way ANOVA indicated no significant differences for treatment (saline versus PTZ) or age factors (young versus middle-age) in the time spent and number of entries in the open arms of the maze (Figure 2A,B). Moreover, two-way ANOVA indicated a significant effect for age factors in the number of entries into enclosed arms (F_1,32_ = 10,3, *p* < 0.005) (data not shown) and the total number of entries on arms (F_1,32_ = 9,84, *p* < 0.005). Subsequent Newman-Keuls tests indicated a decreased number of this parameter in middle-aged rats treated with saline (Figure 2C). Two-way ANOVA also revealed significant differences for age factors when ethologic behaviors such as protected stretch-attend postures F_1,32_ = 5,99, *p* < 0.05) and rearing (F_1,32_ = 10,2, *p* < 0.005) were evaluated. Subsequent Newman-Keuls post-hoc tests indicated that kindled middle-aged rats were prone to reduce the number of protected stretch-attend postures and rearing behaviors (Figure 2D,E) when compared to kindled younger rats. The number of entries into open arm´s extremities was not significantly altered by treatment or age factors (Figure 2F).

### 2.3. Object Recognition Memory Following Kindling Induction in Middle-Aged Rats

**Figure 3:**
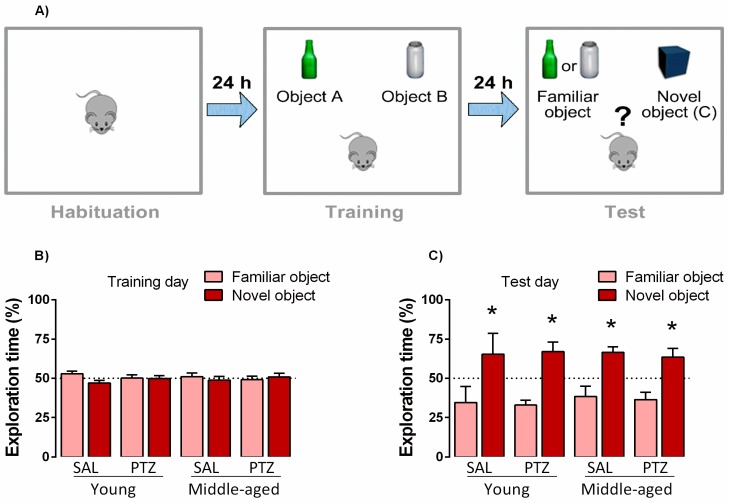
Effects of kindling on cognitive performance of middle-aged rats on object-recognition task. Experimental timeline (**A**). Values are represented by the mean ± S.E.M., followed by Student’s *t*-test when required during training (**B**) and test (**C**) days. * *p* < 0.05 in the comparison between familiar and novel objects (*n* = 8–12/group).

The object recognition task was used to evaluate the effects of kindling on the long-term recognition memory of young and middle-aged rats (Figure 3). The Student’s *t*-test revealed no significant differences in the exploration time of objects A and B during the training phase, indicating that all groups explored both objects equally. During the test phase, both saline- and PTZ-treated young animals spent much more time exploring the novel object than the familiar one (t = 3.02; *p* < 0.005 and t = 2.08, *p* < 0.001; respectively), and similar results were observed for saline- and PTZ-treated middle-aged rats (t = 3.57; *p* < 0.004 and t = 2.69, *p* < 0.002, respectively). These findings indicate that all groups were able to recognize the familiar object.

### 2.4. Hippocampal MAPKs Levels Following Kindling Induction in Middle-Aged Rats

**Figure 4:**
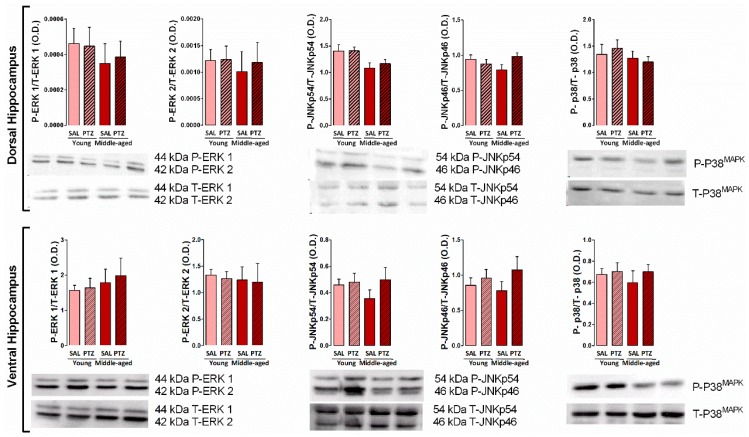
Effects of kindling on expression of mitogen-activated protein kinases (MAPKs) (ERK 1/2, JNK p54/p46, p38) into the dorsal and ventral hippocampus of middle-aged rats. Values are represented by the mean + S.E.M. followed by two-way ANOVA (*n* = 6/group).

To determine the effects of kindling on the levels of MAPKs into the dorsal and ventral hippocampus of middle-aged rats, we measured the immunocontent of both phosphorylated (active) and total forms of ERK 1/2, JNK p54/p46, and p38^MAPK^ protein. Two-way ANOVA revealed no significant effects of the main factors (treatment and age) in the phosphorylation level and total content of ERK 1/2, JNK p54/p46, p38^MAPK^ protein into the dorsal hippocampus. Moreover, no significant changes of these proteins were observed into the ventral hippocampus (*p* > 0.05, Figure 4).

## 3. Discussion

To the best of our knowledge, this is the first report of the effects of epileptogenesis on emotional, cognitive, and neuroplasticity-related hippocampal cell signaling in middle-aged rats. The present findings show that repetitive injections of subconvulsive doses of PTZ differently alter seizure resistance and duration in young and middle-aged rats during the kindling protocol, besides not to impair emotional and cognitive performance, nor the hippocampal MAPKs (ERK1/2, JNK p54/p46, p38^MAPK^) phosphorylation and total content of these enzymes. Notably, despite behavioral age-related differences during epileptogenesis, young and middle-aged rats perform equally after kindling, indicating the existence of shared effects in controlling hippocampal-dependent tasks and signaling cascades following the insult.

Overall, the investigation of acute seizures and epilepsy in aged animals is scarce in the literature [4]. Our results concerning the induction of kindling demonstrated age-dependent variations between 3 and 12 months old rats, these last categorized as middle-aged animals [13]. The kindling protocol developed earlier in young animals when compared to middle-aged rats. Further, a higher resistance to PTZ kindling was observed in middle-aged rats, whereas young kindled rats presented a higher mortality rate (young: 33.3%; middle-aged: 20%). Our results are in agreement with previous studies showing age-related differences in the susceptibility to chemical-induced kindling [6,14] and electrical stimulation of the amygdala [15] or hippocampus [7]. For instance, it was reported that PTZ-kindling was reached earlier in mice aging two months old as compared with 6 and 12 months old. Moreover, the resistance to kindling was increased in accordance with the age of animals, with higher resistance observed in 12 months old (30%) followed by 6 months old (10%) and 2 months old animals [14]. Additionally, 18 and 24 months old animals do not developed generalized clonic or tonic–clonic seizures when injected with subconvulsant doses of PTZ (35 mg/kg; i.p.) on alternated days [6].

In our study, kindling or aging factors did not affect the classical parameters of anxiety-like behaviors, including the time spent and number of entries on open arms of the maze. However, both middle-aged (kindled and vehicle) groups showed a significant decrease on protected stretch-attend postures behavior when compared to respective young groups, suggesting a decrease on emotional reactivity to conflict approach/avoidance and a reduction of defensive behavior. Further, the chronic administration of PTZ is reported to induce opposite effects on anxiety-like behaviors [16,17]. Here, PTZ-kindling did not affect the classical parameters of anxiety-like behaviors evaluated in the EPM test. These observations partially corroborate with Becker and colleagues, where fully PTZ kindled rats behaved similar as control animals in the EPM test [18]. On the other hand, it was also reported that PTZ-kindled rats exhibit anxiolytic-like responses in the social interaction and EPM tests carried out after the last administration of PTZ [16,17]. Further, evidence of anxiety-like behavior has been reported following the electrical kindling of the amygdala, an animal model analogous to temporal lobe epilepsy of humans [19,20]. Thus, it is possible to speculate that a previous exposure to an anxiogenic stimulus—such as PTZ administration—may result in habituation when facing an anxiety-provoking stimulus, which possibly reflects these results.

Further, both middle-aged groups (PTZ-kindled and vehicle) also exhibited a significant decrease in locomotion and exploratory behaviors, with a decline of total entries of arms and rearing in the EPM test and general ambulation during the habituation of object recognition task (data not shown) when compared to the other groups. These responses could be partly explained by the physiological deterioration of the musculoskeletal and locomotor system, and/or a decrease of novelty-seeking behaviors that commonly occurs along aging [21]. Also, we cannot discard the involvement of other key brain regions that can modulate both seizures and anxiety-like behaviors. In particular, the amygdaloid and thalamic nuclei and the substantia nigra, which is deeply affected during PTZ-kindling and is associated with significant changes of GABAergic neurons and BDZ-binding [22]. In this line, the mild alteration of ethologic behaviors, such as protected stretch-attend postures and rearing, may be altered by the voluntary movement centers that are also responsible for seizure spreading [23].

Accordingly, neither age or kindling affected the performance of animals in the one-trial version of the object recognition memory task, which is a validated test on the studying of non-spatial memories, based in the natural tendency of animals to explore novel objects [24]. Our findings are consistent with the literature where recognition memory, evaluated in the object recognition task, was unaffected in aged rats [25,26]. It is important to point out that the duration of interval between training and choice trials (test) carried out in these studies was about 1 h or less. Here, the inter-trial interval duration was increased to 24 h, taking into account the previously reported age-related deficits in the object recognition behavior [27]. Conversely, our results showed that all groups performed similarly during training and test days, where all of them prefer to explore the novel object. To the best of our knowledge, only a few studies have reported age-dependent learning deficits in PTZ- [6,14] and electrical-kindled rats [7]. In fact, PTZ-kindled aged rats presented a significantly impaired learning and memory performance as compared to naïve, but not in an age-dependent manner. Grecksch and colleagues [6] reported that only 24 months old kindled rats displayed an impaired learning and memory task, whereas, another study demonstrated a “U” shape response on cognitive decline, showing that adult rats present a better performance when compared with younger and older rats with severe learning deficits [14]. Indeed, we demonstrated that PTZ-kindling did not affect cognitive performance assessed in the object recognition task. Despite the methodological differences, our results are in agreement with Lamberty and Klitgaard [28], since spatial reference and working memories assessed in the Morris water maze test were not altered following PTZ-kindling. On the other hand, it has been shown that both PTZ- and electrical-kindling of the amygdala evoke marked cognitive dysfunction as assessed in the Morris water maze and fear conditioning tasks [18,29]. Therefore, it is possible to propose that different degrees of cognitive impairment may be related to distinct animal models of epilepsy, thus requiring further studies to elucidate this issue.

Importantly, clinical studies addressing cognition in epilepsy have produced distinct results, with suggestions of impaired cognitive ability [30], a stable neuropsychological functioning [31], or a progressive cognitive decline with ongoing seizures, with recovery from deficits when seizures cease [32]. Data from human subjects suggest substantial cognitive deficits at the onset of epilepsy, which stabilize during the first 5–10 years of epileptic life [33]. Overall, these clinical studies support the idea that the progressive epilepsy-associated decline in cognitive function is strongly linked to uncontrolled seizures. Considering that, the PTZ-kindling model does not lead to mesial temporal lobe epilepsy, but to generalized seizures with focal onset. Thus, it is likely that this model does not cause typical features that are observed in epilepsy as other translational animal models of epilepsy [34]. Thus, it is possible that our results are at variance with the literature due to different methodological aspects.

In addition, different animal models of epilepsy (i.e., kainate, pilocarpine, PTZ- and electrical-kindling of amygdala) may promote intense or mild neuronal injury, suggesting that neuronal injury should be a crucial factor related to cognitive impairment, particularly in the mesial temporal lobe epilepsy [34]. In humans, monkeys, and rodents, recognition memory is affected by damage in structures of the mesial temporal lobe, including the hippocampus, and adjacent entorhinal and parahippocampal cortices [35]. In this sense, it is possible that kindling and aging does not result in cognitive deterioration sufficient to change the performance in a hippocampus-dependent learning and memory paradigm.

Importantly, we cannot exclude the effects of aging and kindling in neurogenesis and their modulatory role in seizure susceptibility and cognitive performance outcome. The increased expression of the growth-associated protein (GAP-43) in the hippocampus was coincidently associated with an increased seizure susceptibility of rats that had undergone kindling besides its transient modulation across aging. Interestingly, resembling young rats, the middle-aged rats showed similar expression of GAP-43 mRNA in the hippocampus, whereas GAP-43 protein was higher in older rats. These findings highlight a retained neuronal regenerative potential of middle-aged rats following an insult, unlike older rats, which completely lost this ability [8,36,37]. These results may contribute to the lack of effect on cognitive performance once brain plasticity may be not significantly impaired following kindling in middle-aged rats.

Importantly, the MAPK cascade is essential for synaptic plasticity and learning. In the hippocampus, three different MAPK subfamilies, extracellular signal-regulated kinase 1/2 (ERK1/2), p38 MAPK, and c-Jun NH2-terminal protein kinase (JNK), selectively regulate activity-dependent glutamate receptor trafficking during long-term potentiation (LTP) and/or long-term depression (LTD), which together are considered the most promising candidate as a cellular mechanism underlying learning and memory [10]. The present study showed no changes in the expression and activity (phosphorylation level) of hippocampal MAPKs (ERK1/2, JNK p54/p46, p38^MAPK^) among the four experimental groups, independent of aging and kindling. There is good evidence that MAPK/ERK and the cAMP response element-binding protein (CREB) may be part of a signaling cascade involved in the regulation of synaptic plasticity processes required for object recognition memory [38] as well as for other forms of learning and memory [39,40]. For instance, it is reported that manipulations that disrupt the activity of these biochemical effectors—such as inhibiting MAPK/ERK kinase (MEK) cascade—produce a deficit in the consolidation or expression of memory [38,40]. Our findings indicated that there is no significant differences in the immunocontent of either phosphorylated or total protein ERK 1/2, JNK p54/p46, p38^MAPK^ in the hippocampus, independent of aging or the pathological status of rats, which also exhibited similar cognitive performance in the object recognition task. It is important to highlight that the functional integrity of the mesial temporal lobe is essential for object recognition memory processing [41]. Thus, it is possible that the aging and kindling process are not able to produce an enduring damage on the hippocampus capable to modify the immunodetected content and the activity of MAPK subfamilies neither affect the emotional and cognitive performance of rats in the object recognition task.

## 4. Material and Methods

### 4.1. Animals

Young and middle-aged male Wistar rats (3 or 12 months old, 200–300 g or 400–480 g, respectively) were housed in groups up to four per cage and kept in a room with controlled temperature (22 ± 2 °C) and a 12-h light/dark cycle (lights on at 07:00 a.m.) with free access to food and water, except during the experiments. Rats were allowed to adapt to the laboratory conditions for at least one month before the experiments. Behavioral experiments were carried out during the light phase of the cycle (between 13:00 and 18:00). Experiments were conducted in accordance with international standards of animal welfare recommended by the National Institutes of Health Guide for the Care and Use of Laboratory Animals, with experimental protocols being approved by the Committee for Ethics in Animal Research of the Federal University of Santa Catarina (CEUA-UFSC #PP00893/2014). The minimum number of animals and duration of observation required to obtain consistent data was used.

### 4.2. Drugs and Treatments

Pentylenetetrazole hydrochloride (PTZ, a GABA-A receptor antagonist; Sigma-Aldrich Co., St. Louis, USA, range of 35 to 50 mg/kg) was freshly dissolved in saline (NaCl 0.9%), which was also used as control solution. Drugs were injected intraperitoneally in a volume injection of 1 ml/kg. All of the doses used here were taken from previous studies [6,12].

### 4.3. Experimental Procedures

#### 4.3.1. Induction of Kindling

**Figure 5:**
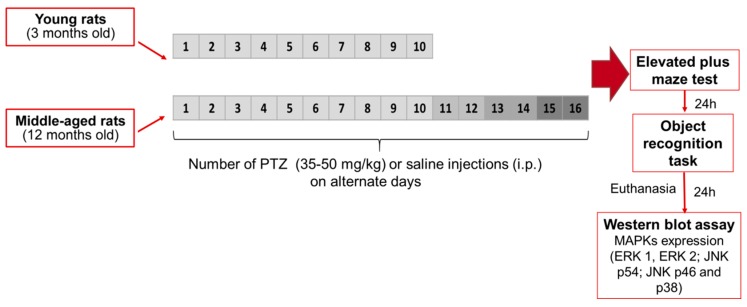
Outline treatment and experimental schedule showing the PTZ kindling model of epilepsy. Twenty-four hours following the treatment protocol with administration of saline solution (Sal, 0.9%) or pentylenetetrazole (PTZ, 35, 40, 45, or 50 mg/kg), animals were evaluated with a 24 h-interval between tests in the elevated plus-maze and object recognition task. The hippocampus were dissected for Western blotting analysis 24 h following behavioral tests.

Both young and middle-aged animals were injected with subconvulsive doses of PTZ at 48 h intervals during 20 days (young rats, *n* = 17) or 32 days (middle-aged rats, *n* = 11). Due to the increased resistance of middle-aged animals in fully developing kindling during the experimental protocol, doses of PTZ were gradually elevated following the 10th injection (additional 5 mg after each 2 injections) until reaches the kindling status (two consecutive experimental days with seizures stages 4 or higher) or 16 injections. Immediately after injection the rats were placed in a plastic chamber (15 × 30 × 42 cm) and convulsive behaviors were scored during 5-min periods during 30 minutes, according to the criteria of the modified Racine’s scale [42]: Stage 0, lack of any apparent seizure activity; Stage 1, sudden immobilization; Stage 2, stereotypy (ear and facial twitching); Stage 3, facial, vibrissal and forelimb clonus (short myoclonic jerk) and/or wet dog shake behavior; Stage 4, sporadic forelimb clonus with kangaroo posture; Stage 5, clonic and/or tonic seizures with posture maintenance; Stage 6, generalized tonic-clonic seizures with loss of posture tone, and turning onto the side and seizures characterized by rearing and falling; and, Stage 7, death within 30 min. PTZ administration was withdrawn after establishment of kindling status and subsided by saline injections until the end of the protocol. Similarly, control groups of both ages were treated with saline solution (0.9% NaCl) and underwent similar manipulation (see protocol design in Figure 5).

#### 4.3.2. Elevated Plus-Maze (EPM)

The EPM apparatus was made of wood and consisted of two opposing open arms (50 × 10 cm) and two opposing enclosed arms (50 × 10 × 30 cm), all facing a central platform (10 × 10 cm), elevated 40 cm from the floor. To prevent falls, the open arms were surrounded by a 1-cm high acrylic rim. The apparatus was placed in a small closed room lit by a white light that provided 30 lux in both the open and closed arms. Each rat was used only once and individually placed on the central platform facing an enclosed arm. The frequency of entries into either open or enclosed arms, as well as the time spent in each arm type, were recorded (in seconds) for 5 min. Ethological parameters such as unprotected head-dipping, open-arms end activity, and protected stretch-attend postures were also recorded to increase the sensitivity of the test [43]. The apparatus was cleaned with 10% ethanol solution between sessions.

#### 4.3.3. The Object Recognition Task

The object recognition task was conducted in an open field arena (50 × 50 × 50 cm) made of wood in a room lit by a white light that provided 30 lux and few environmental cues. The objects presented had different colors and shapes, but similar dimensions. Each object was glued to the floor of the open field with adhesive tape being positioned about 10 cm from the walls of the apparatus and 20 cm from other objects, and the positions of the objects were counterbalanced in each session. The experimental protocol of the object recognition task consisted of three distinct phases: habituation, training, and test. Prior to the test, all of the animals were habituated to the arena by allowing them to freely explore it for 30 min without the presence of any object. On the second day (training), all rats were exposed to two different objects (A and B) for 10 min. Twenty-four hours later (test), the rats were exposed in the open field for 10 min to one of the objects that were previously presented during the training phase (object A or B) associated with a novel object (object C).

The time spent by the animals exploring each object was recorded by a video system and analyzed later. Exploration of an object was defined when the rat directed the nose to the object at a distance equal to or less than 2 cm, and/or touching it with the nose and/or forepaws. The time spent exploring each object was expressed (in seconds) as a percentage of the total exploration [44]. The apparatus was cleaned with 10% ethanol solution between sessions.

#### 4.3.4. Western Blotting

All animals were euthanized twenty-four hours following the last behavioral evaluation. The brains were excised from the skull and hippocampi dissected (4 °C), placed in liquid nitrogen, and then stored at −80 °C. Briefly, samples were mechanically homogenized 1:4 (w/v) in 400 μL of Tris 50 mM pH 7.0, EDTA 1 mM, NaF 100 mM, PMSF 0.1 mM, Na_3_PO_4_ 2 mM, Triton X-100 1%, glycerol 10%, and Sigma Protease Inhibitor Cocktail (P2714), followed by incubation for 10 min in ice. Lysates were centrifuged (10,000× *g* for 10 min, at 4 °C) to eliminate cellular debris. The supernatants were diluted 1/1 (v/v) in Tris 100 mM pH 6.8, EDTA 4 mM, SDS 8%, and boiled for 5 min. Thereafter, samples were diluted (40% glycerol, 100 mM Tris, bromophenol blue, pH 6.8) in the ratio 25:100 (v/v) and β mercaptoethanol (final concentration 8%).

The same amount of protein (60 μg per lane) for each sample was electrophoresed in 10% SDS–PAGE minigels and transferred to nitrocellulose membranes by using a semidry blotting apparatus (1.2 mA/cm^2^; 1.5 h). The membranes were blocked with 5% skim milk in TBS (Tris 10 mM, NaCl 150 mM, pH 7.5). The total content and phosphorylation of MAPKs (ERK 1/2, JNK p54/p46, and p38MAPK) were detected after overnight incubation with specific antibodies diluted in TBS-T containing BSA 2% in the dilution of 1:5000 (anti-phospho ERK1/2, anti-phospho JNK p54/p46, anti-total JNK p54/p46), 1:40,000 (anti-total ERK1/2); 1:10,000 (anti-phospho p38MAPK, anti-total-p38MAPK). Moreover, the membranes were incubated for 1 h at room temperature with horse radish peroxidase (HRP)-conjugated anti-rabbit (1:5000) or anti-mouse (1:5000) secondary antibodies for detection of phosphorylated sites or total form, respectively. The membranes were incubated with a chemioluminescence substrate (LumiGLO) to cause the reactions. All of the blocking and incubation steps were followed by three washings of 5 min with TBS-T (Tris 10 mM, NaCl 150 mM, Tween-20 0.1%, pH 7.5). All of the membranes were incubated with mouse anti-β-actin (Santa Cruz; 1:2000) antibody to verify that equal amounts of proteins were loaded on the gel. The phosphorylation level of proteins was determined as a ratio of OD of phosphorylated band/OD of total band. The bands were quantified using the Scion Image^®^ software.

### 4.4. Statistical Analysis

Values are expressed as means ± S.E.M or median ± interquartile range. Data of experiments were analyzed by two-way ANOVA (age and treatment as independent factors), followed by the Newman-Keuls post-hoc test for multiple comparisons when appropriate or by Kruskal-Wallis ANOVA or Mann-Whitney U test when non-parametric analysis was required. Differences were considered significant at *p* ≤ 0.05. All of the tests were performed using the software Statistica^®^ (StaSoft Inc., Tulsa, OK, USA) version 8.0, and graphs were drawn with the software GraphPad Prism^®^, version 5.0.

## 5. Conclusions

In summary, despite the age-related differences observed during epileptogenesis induced by kindling, both the young and middle-aged rats presented a similar performance in discriminative learning and emotional tasks besides not to differ the content and activity of hippocampal MAPKs, denoting that the analogous shared effects are evoked even following distinct epileptogenic mechanisms. Additional investigation must be carried out to better explore the electrophysiological and biochemical mechanisms underlying these responses, as well as the effects observed long after kindling.
